# Some Remarks Concerning the Use of Mercury in the Treatment of Syphilis

**Published:** 1892-02

**Authors:** F. A. Rietema

**Affiliations:** Rotterdam


### SOME REMARKS CONCERNING THE USE OF MERCURY IN THE TREATMENT OF SYPHILIS.*

BY DR. F. A. RIETEMA, ROTTERDAM.

Translated for THE JOURNAL.

In the effort, at present noticeable, to bring a number of new remedies into the therapy of syphilis, it may not be amiss to inquire:

(1) If the need of new remedies is actually so great as one would believe, considering the large addition; and

(2) Whether the old remedies do not meet every indication in syphilis when properly employed.

*From Monatshefte fur Praktische Dermatologie, Band XII, No. 12.—H.

In answer to these questions, we will now mention the methods of treatment customary throughout middle Europe. The inconveniences of the oldest of all methods of treatment (inunctions) direct us to the effort of trying to find other and better methods if possible, and even to a certain prejudice against this method, in consequence of which one looks at its defects through a magnifying glass while he overlooks or condones those of other plans. Is it fair to object to the inunction method that one does not know how much of the mercury "rubbed in" is taken up by the system? Is it just for Rossbach to say? "However, the simplest and best rule, that the physician should know how much of a strongly poisonous remedy is introduced into the system, cannot be followed with reference to the gray ointment, or how much of that taken up is rendered available through oxidation. And if one would reply that the greater part of the mercury is wasted in inunctions and only the smallest portion has effect, we might ask if that would be reason to order a remedy with directions to pour out ninety-nine parts and to take one part? No; for these objections hold also for the other methods, save hypodermic injections with soluble mercurials."

Does any one know what proportion of the at present so esteemed injections with emulsions of insoluble mercurials or mercury in substance enters the circulation? By no means. That which is taken up from the injections withdraws itself in every direction, whether the quantity $x$ is taken up in one, ten or one hundred days, one does not know; nor, much the less, whether $x$ is taken up in a space of $y$ days; and just here is not only the defective, but also the dangerous side of the method of injecting insoluble mercurial salts into the organism.

Exactly because of that uncertainty as to absorption, one is exposed to the fear that quantities might be taken up in a short time which would produce extremely unpleasant symptoms, as stomatitis and intestinal disorders. If that happens, one stands completely helpless against it; while one can stop the mercury in all other methods, he cannot here, because these is a depot in the system from which absorption is constantly taking place. Excision of the site of injection will be almost an impossibility.

According to our opinion, those dangers make the method unconditionally objectionable (even if they were actually as rare as some prefer to specify, although far from all unfavorable occurrences reach the public). We should like to place their chance of occurrence at nothing, even if the other methods fell far short of its usefulness, which is by no means the case; for, at present, "the greater therapeutic effect" of injections with insoluble mercurial salts seems to become more problematic the longer they are used, but there are observers who think they have noticed the number and severity of relapses increased under this method. And even if one should doubt this, it is certain that the method does not act more rapidly, its single advantage being the convenience of its use. If, however, two bridges cross a river, one of which, though short and easy to cross, threatens every moment to fall, while the other, though longer and more difficult, is of far greater strength than the first, no novice, indeed, acquainted with the characteristics of both would dare to prefer and recommend the first. Certainly, every one would choose the second. That which is not allowed of the "novice" would certainly not be permitted in the physician.

I am, therefore, convinced that, in any event, we must give up the method which promises so little. It is now the fashion, and "the gods battle in vain against fashion." Here it must not be forgotten that the introduction of a new, insoluble mercurial salt for injection is an excellent means of getting a little reputation for learned gentlemen who have, as yet, none.

Theoretically there is nothing to object against the use of soluble mercurial salts. The procedure is rational; the intestinal canal is not affected; it permits accurate dosage; is as little fraught with the danger of producing abscesses if rightly employed as the use of the insoluble, and with it one can stop the mercury at any moment without fearing the cumulative effect of that previously injected, as in the case of the insoluble. The single objection to this method is that the patient must visit the physician daily or at least every other day. But this objection is so great in private practice that it forms the chief drawback in the use of this intrinsically valuable measure.

As regards the inunction method, a hundred years' experience has proven its value, and where, in medicine, Practice speaks Theory must be silent. Our view is that the inunction method, when well applied, is one of the best. Proper application alone leaves much to be desired. In hospitals and private institutions, where one has a well-trained staff and under his constant personal observation, the method acts excellently. It is different in private practice where the patient rubs himself, and, as a rule, the results are miserable. A common fault is that one does not rub long enough, and, further, the patient exercises too severely, producing sweating, which renders illusory any small effect which would be obtained from the too brief rubbing. In private practice we consider the inunction method but little capable of results.

We have believed ourselves able to establish our unfavorable judgment concerning the injection of insoluble mercurial salts, and we have seen that the, in every way proper, injections with soluble (mercurial) salts meet the difficulties of practice. It remains now to answer the question whether the yet to be mentioned method, the internal employment of mercurial preparations, meets all desirable requisites, both in theory and practice.

Among the Germans, especially, there exists a prejudice against this method, the cause of which is not easy to demonstrate. We order *per orem* the greater number of all medicines. Practice has taught how great the quantity shall be for man in any special case. For instance, no one has to estimate how much to give when quinine, digitalis, antipyrin, salicylate of soda, etc., are to be administered by the mouth, and yet one is quite content with results obtained. However, as soon as the procedure extends to the mercurial preparations, one must estimate somewhat, and does not seem to think or deny that a remedy, which has been used by millions of Romanish people for a hundred years, must possess undoubted peculiarities which justify that procedure, or it would be to deny all judgment to the Romans, and we should not like to do that. On the contrary, we hold this method (presupposing its rational employment) directly available everywhere as a curative procedure which fulfills every reasonable expectation.

One may say in general that there is expected of a remedy:

(1) With its use the symptoms vanish the most quickly.

(2) That either no, or the slightest possible, unpleasant appearances occur, and especially no effects which render questionable the results of its use.

Are there mercurial combinations which, used internally, meet both these requirements? I believe I can answer in the affirmative with complete truth.

In the round of practice the majority of physicians come to use special combinations, which gives the not to be underestimated advantage of knowing better what they may expect from their treatment in each particular case, over those who order the numerous mercurial preparations in succession. We employ preferably the tannate of mercury, corrosive sublimate or calomel—the latter especially in practice among children. In the first year and a half following infection we rarely use iodine and mercury combinations. The following is the plan of treatment:

The treatment is begun as soon as the symptoms of general infection show themselves on the skin. For reasons, the detailing of which would take us too far, we are absolute opponents of the method of beginning treatment while the "hard ulcer" exists alone, without further evidences of syphilis. As soon as the "roseola" appears the patient begins taking (about) one and a half grains of tannate of mercury three times a day, immediately after meals; the *ulcus durum* alone is treated with mercurial plaster, while the treatment is appropriately modified in cases where soft chancre complicates the hard.

The patient is instructed to keep the mouth scrupulously clean; nevertheless we have him avoid the too frequent brushing of the teeth and gums, which we permit at most only once a day, but advise the patient to rinse out his mouth with water immediately after eating anything. We believe the entirely too energetic brushing of the teeth less advantageous because we have noticed that patients who are not accustomed to cleaning their teeth then get the mucous membrane of the mouth into a state of irritation which predisposes to stomatitis. When symptoms disappear fairly rapidly we continue the above treatment at least thirty-five days, because we believe it highly important that the first course of treatment should be as thorough as possible. If a mild salivation arises during that time it must be treated in the usual way. If none occurs the dose is somewhat increased until it does occur, as we believe slight salivation is evidence that the system is saturated with the drug.

If the symptoms (of syphilis) disappear in just thirty-five days the treatment must be kept up for fifty-three days, as we follow, the rule that it must continue one and a half times as long as the time required to produce complete disappearance of the symptoms. The somwhat increased stools, usual at first, soon return to normal or simply remain a little "watery" while diarrhœa seldom occurs and is easily controlled. We have not yet observed any other unpleasant symptoms.

Relapses are treated either with tannate of mercury or with the bichloride. With the latter the following is the plan: Of a solution of bichloride 1-1000 in distilled water, we have the patient take twenty-five minims ($\frac{1}{40}$ gr.) diluted in a glass of water, three times a day, immediately after meals, and increasing the dose, according to slowness or rapidity of subsidence of symptoms, to double, hence $\frac{1}{20}$ gr. of the bichloride.

I have never noticed any stomach or intestinal trouble in connection with this treatment, the slightly increased and easily controlled stools being the only effect. That corrosive sublimate has got the reputation of easily causing derangement of the stomach arises from the manner in which it is often used. However, one finds recipes recommended in the latest works upon syphilis, in which the drug is provided in pill form and increased doses, and this while twelve years ago we could read in Nothnagel's and Rossbach's book—after they had shown that sublimate undiluted was a corrosive substance: "The opinion that the stomach gradually gets accustomed to large doses of corrosive sublimate is as nonsensical as would be the belief that the animal body could finally get so as not to be burned with fire if one only gradually increased the degree of the heat. The animal and human body becomes accustomed to no corrosive substance through increasing dosage. A certain dose and concentration will always produce corrosion whether or not the organism had previously received smaller doses."

We are very well satisfied with the results of the treatment detailed. Self-evidently, complications, which however are rare, would receive the proper attention, also the (not very common) cases where local affections demand local treatment. Affections of the scalp and loss of hair are treated with the following pomade: White precipitate, ʒi; corrosive sublimate, grs. 3; vaseline and lanolin, aa ℥iiss; oil of rose, 5 drops, after the scalp has been thoroughly cleansed with a solution of bicarbonate of soda, which at the same time acts favorably upon the pityriasis capitis (scaling) sometimes complicating the trouble. No mercury is given during the intervals of freedom from symptoms, with this reservation, however, that should no relapse occur (a truly rare, but yet an event which may happen), three "courses of treatment," at least, should be gone through. As it will take up too much space we will not go into the treatment of the later so-called tertiary stages.

Considering what has been said above, we perceive that the inunction method is very good where the patient's circumstances permit its use; likewise the injections of soluble mercurial salts, especially in the presence of intestinal disorder; that, on the other hand, where the digestive organs are normal, the internal treatment is far to be preferred to inunctions as used in private practice. Our experience shows that a properly directed internal treatment is of equal value with properly used inunctions or injections with soluble mercurial preparations, and is of far greater value than the to us always objectionable injections with insoluble mercurial preparations.

---

THE many former students of the Charleston Medical College will learn with regret of the death of Dr. R. A. Kinloch, which occurred December 23d. Dr. Kinloch was a gentleman of the highest character, an excellent teacher and for years one of the most prominent of Southern surgeons.

